# Cx43 Channel Gating and Permeation: Multiple Phosphorylation-Dependent Roles of the Carboxyl Terminus

**DOI:** 10.3390/ijms19061659

**Published:** 2018-06-04

**Authors:** José F. Ek-Vitorín, Tasha K. Pontifex, Janis M. Burt

**Affiliations:** Department of Physiology, University of Arizona, P.O. Box 245051, Tucson, AZ 85724, USA; tasha@email.arizona.edu (T.K.P.); jburt@email.arizona.edu (J.M.B.)

**Keywords:** casein kinase 1, phosphorylation, channel gating, gap junction permeability, arrhythmia

## Abstract

Connexin 43 (Cx43), a gap junction protein seemingly fit to support cardiac impulse propagation and synchronic contraction, is phosphorylated in normoxia by casein kinase 1 (CK1). However, during cardiac ischemia or pressure overload hypertrophy, this phosphorylation fades, Cx43 abundance decreases at intercalated disks and increases at myocytes’ lateral borders, and the risk of arrhythmia rises. Studies in wild-type and transgenic mice indicate that enhanced CK1-phosphorylation of Cx43 protects from arrhythmia, while dephosphorylation precedes arrhythmia vulnerability. The mechanistic bases of these Cx43 (de)phosphoform-linked cardiac phenotypes are unknown. We used patch-clamp and dye injection techniques to study the channel function (gating, permeability) of Cx43 mutants wherein CK1-targeted serines were replaced by aspartate (Cx43-CK1-D) or alanine (Cx43-CK1-A) to emulate phosphorylation and dephosphorylation, respectively. Cx43-CK1-D, but not Cx43-CK1-A, displayed high Voltage-sensitivity and variable permselectivity. Both mutants showed multiple channel open states with overall increased conductivity, resistance to acidification-induced junctional uncoupling, and hemichannel openings in normal external calcium. Modest differences in the mutant channels’ function and regulation imply the involvement of dissimilar structural conformations of the interacting domains of Cx43 in electrical and chemical gating that may contribute to the divergent phenotypes of CK1-(de)phospho-mimicking Cx43 transgenic mice and that may bear significance in arrhythmogenesis.

## 1. Introduction

Gap junction channels made of Connexin (Cx) proteins support the propagation of electrical impulses from cell to cell, and are therefore indispensable for synchronic heart contractions. Cx43, the most widely distributed cardiac Cx and abundant in all four chambers [1], can compensate for atrial Cx40 deficiency and prevent fibrillation [2]. However, replacing or supplementing Cx43 with other Cx isotypes promotes arrhythmia vulnerability [3,4]. These data suggest that Cx43 is optimally endowed to support cardiac conduction. While such uniqueness may derive from Cx43 amenability to regulation by multiple kinases [5,6], the functional ramifications of such regulation remain poorly defined. To address this deficit, we aim to outline the operational profiles of Cx43 modified by specific phosphorylation events.

In normal heart, Casein Kinase 1 (CK1) phosphorylates serines 325, 328 and 330 of the Cx43 protein residing at gap junctions (GJs) of intercalated disks (ID) [7,8]. During acute ischemia or chronic pressure overload hypertrophy (induced by transverse aortic constriction, TAC), phosphorylation of these serine residues is greatly reduced, while total Cx43 protein decreases at the ID and increases at the lateral sides of cardiomyocytes [8,9]. This gap junction remodeling (GJR) alone might slow impulse propagation and contribute to arrhythmia. In accordance, spironolactone, an aldosterone antagonist with beneficial effects in patients with heart failure [10], prevents dephosphorylation of the CK1 sites and reduces or reverses pathological GJR [11]. These data suggest that reversible CK1-phosphorylation is a pivotal regulatory event that establishes the fate of Cx43 protein/channels during the transition from physiological to pathological states. In agreement, hearts from transgenic mice expressing a CK1-dephospho-mimicking mutant of Cx43 (substitutions S325,328,330A, named S3A) exhibit enhanced GJR and high propensity to arrhythmias after ischemia or TAC-induced hypertrophy. A converse CK1-phospho-mimicking mutant (substitutions S325,328,330E, named S3E) had opposite effects, that is, hearts were resistant to arrhythmia induction and to pathological GJR after ischemia or TAC [9]. Despite the clinical interest of such Cx43 (de)phosphoform-linked cardiac phenotypes their mechanistic (functional) bases remain unknown.

We hypothesized that differences in channel gating/permeability, linked to the phosphorylation status of Cx43, contribute to the cardiac phenotypes of S3A and S3E mice. Therefore, we assessed gating and permeability of mutant Cx43-S325,328,330D (here dubbed Cx43-CK1-D), where aspartates supply the charge effect of phosphorylation, and mutant Cx43-S325,328,330A (dubbed Cx43-CK1-A), where non-phosphorylatable alanines provide a stable “dephosphorylated” state. While both mutants displayed properties typical of wild-type Cx43 (Cx43WT), they also showed unexpected properties that challenge current models of channel function and may be significant for the cardiac phenotypes of the (S3A, S3E) transgenic mice, as well as for the pathophysiology of cardiac ischemia and arrhythmogenesis.

## 2. Results

### 2.1. Cx43-CK1-D Displays Stronger V_j_-Sensitivity than Cx43-CK1-A

**Figure 1:**
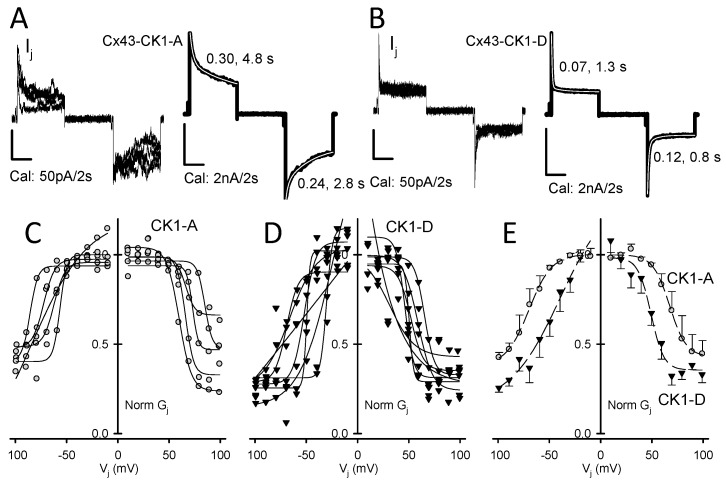
Cx43-CK1-D displays stronger *V_j_*-sensitivity than Cx43-CK1-A. (**A**) Five individual *I_j_* responses to *V_j_* = ±80 mV pulses (left) and sum (right) of 38 similar traces from Cx43-CK1-A cell pairs; (**B**) Five individual *I_j_* responses to *V_j_* = ±80 mV pulses (left) and sum (right) of 30 similar traces from Cx43-CK1-D cell pairs. For (**A**,**B**), tau values of *I_j_* inactivation (2nd order exponential decays) are shown and the fits (white) displayed over the corresponding sum traces; (**C**) *V_j_*-dependence of G_j_ from individual experiments in Cx43-CK1-A (*g_j_* = 2.8 ± 1.0; *n* = 5) cell pairs; (**D**) *V_j_*-dependence of G_j_ from individual experiments in Cx43-CK1-D (*g_j_* = 1.7 ± 1.1; *n* = 7) cell pairs. For (**C**,**D**), *g_j_* from each experiment was normalized as described in the Methods and the Boltzmann fits are shown in solid black lines; (**E**) Average *V_j_*-dependence for CK1-A (gray circles) and CK1-D (black triangles) and their corresponding Boltzmann fits (dashed lines). Fast inactivation and *V_0_* values were different between Cx43-CK1-D and Cx43-CK1-A. For fitting parameters, see Table 1, Tables S1 and S2.

The decrease in junctional current (*I_j_*) observed in GJs subjected to large transjunctional voltages (*V_j_*) is called *V_j_*-gating (Figure 1A,B) and is quantified by fitting the ratio of steady state and instantaneous junctional conductance (g_j_^ss^/g_j_^inst^) through a broad *V_j_* range with a Boltzmann function [12,13], with half-maximal response reported as *V_0_* (see Section 4). When measured in rat insulinoma (Rin) cells, Cx43 *V_j_*-gating (Figure S1, Table 1, Tables S1 and S2 and [14]) was similar to that reported for Cx43WT expressed in other cell types [15,16,17]. Cx43-CK1-A showed *V_j_*-gating comparable to Cx43WT (Figure 1; Table 1, Tables S1 and S2). In contrast, Cx43-CK1-D showed a faster response to *V_j_* (Figure 1A,B) and smaller *V_0_* values (Figure 1C,D, Table 1, Tables S1 and S2), revealing a more sensitive closure mechanism than Cx43-CK1-A. This suggests that Cx43 phosphorylation by CK1 enhances junctional *V_j_*-gating, and that highly *V_j_*-dependent gap junction channels (GJChs) populate the intercalated disks.

### 2.2. Cx43-CK1-D and Cx43-CK1-A Display Highly Conductive, V_j_-Sensitive, Channel Transition Amplitudes

As deduced from their unitary conductances (γ_j_) at *V_j_* < *V_0_*, Cx43 GJChs exist in multiple states: closed (C), fully open (O), residually open (R), and several intermediate open states, or substates (S) [16,18,19,20,21,22]. Thus, while only one fully open state may exist, channels can occupy multiple less conductive configurations. These multiple configurations could result from variable structural conformation of the connexin molecules comprising each channel. Transitions between multiple channel states yield a broad range of apparent γ_j_ values that seem to vary with the cell type and with the Cx phosphorylation state (cf. [23,24]). In addition, the presence of other Cxs can modify both the γ_j_ profile and function of predominantly Cx43-comprised GJs [25,26]. It is thus important that recording conditions are standardized and that cells express only the protein of interest. In our current experimental settings [14,22,27,28] and at *V_j_* < *V_0_*, the γ_j_ values for Cx43WT channels are C = 0, O = 100–125, R = 17–35, and S = 55–70 pS. Transitions between these states would produce transition amplitudes of approximately (in pS) 100–125 (O↔C), 17–35 (R↔C), 55–70 (S↔C), 83–90 (O↔R), 45–50 (O↔S), and 35–40 (S↔R). Given the conductance ranges for each transition type, distinguishing each as a distinct peak is generally not possible. *V_j_* > *V_0_* brings GJChs to the residual state (O↔R) from which full closure (R↔C) may occur. Hence, at *V_j_* < *V_0_* Cx43WT displays many transition amplitudes, and at *V_j_* > *V_0_* most likely O↔C, O↔R and R↔C transitions (Figure S2; cf. [16,18,28]). Because Cx43-CK1-D emulates a Cx43 phosphoform found in normoxic hearts (Cx43-p^CK1^), we expected the distribution of channel transition amplitudes of this mutant to resemble that of Cx43WT. In comparison, Cx43-CK1-A emulates a Cx43 phosphoform (Cx43-dp^CK1^) found during hypoxia and displays a decreased incidence of fully open channels when expressed in mesenchymal cells [8].

**Figure 2:**
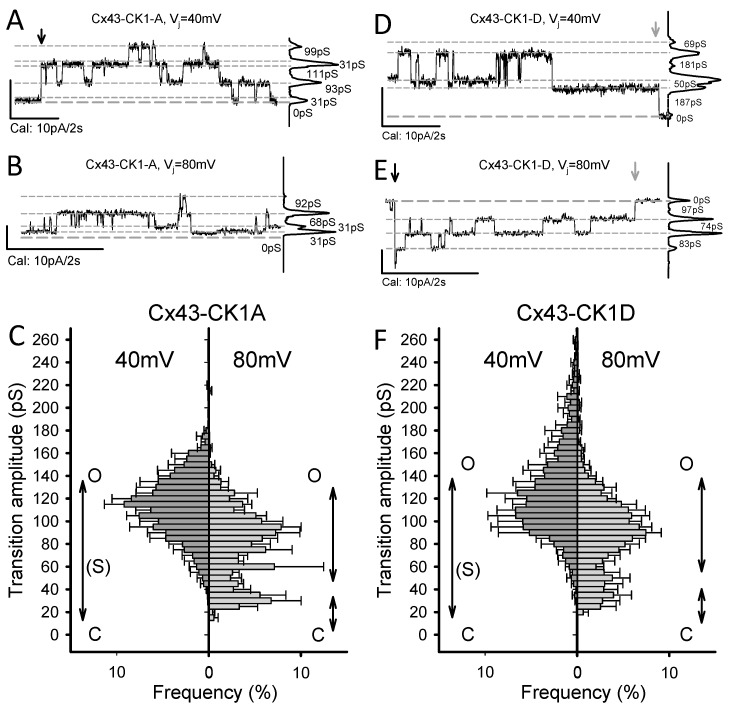
Cx43-CK1-A and Cx43-CK1-D display fully open, *V_j_*-sensitive gap junction channels. (**A**,**B**,**D**,**E**) Illustrative traces of channel activity from Cx43-CK1-A (**A**,**B**) and Cx43-CK1-D (**D**,**E**) expressing cell pairs, at 40 mV (**A**,**D**) and 80 mV (**B**,**E**) transjunctional gradients. For all traces: zero current (long-dashed line) and the most evident *I_j_* levels (short-dashed lines) are indicated; when present, downward arrows mark the beginning (black) and end (gray) of pulses; plots at right are the all-points histogram for the displayed record segment, showing the fraction of time at each *I_j_* level; numbers indicate the conductance change between current levels. Notice that channel transitions often occur between the identified levels. (**C**,**F**) Average transition amplitude histograms at 40 and 80 mV *V_j_* values from Cx43-CK1-A (**C**) and Cx43-CK1-D (**F**). Peak fits indicated by solid black lines. Likely transitions between channel states (see main text for further explanation) are indicated by double arrowed vertical lines. Transition amplitude distributions of Cx43-CK1-D and Cx43-CK1-A differed from each other and from Cx43WT, at both *Vj* values of 40 and 80 mV. However, at *V_j_* = 40 mV, both mutants displayed transitions amplitudes compatible with O↔C and O↔R transitions (if O = 150 pS and R = 30 pS). Transitions larger than 150 pS were documented for Cx43-CK1-D. At *V_j_* = 80 mV, O-R and R-C transitions were more evident for both mutants. However, at both 40 and 80 mV, transitions between closed and levels smaller than fully open states were observed, suggesting substates (S). For each group, the number of experiments (*n*) and measured transitions (N) were respectively, as follows: For CK1-A, 6 and 1867 at 40 mV, 5 and 1080 at 80 mV. For CK1-D, 4 and 1369 at 40 mV, 6 and 1032 at 80 mV.

In Rin cells both mutants showed multiple transition amplitudes, including those consistent with a fully open channel (Figure 2A,B,D,E). Differences in transition amplitudes and distribution with respect to each other and to Cx43WT were documented as follows (Figure 2C,F; cf. Figure S1D): At *V_j_* = 40 mV (<<*V_0_*), main transitions in Cx43-CK1-D were 90–150 pS, and in Cx43 CK1-A, 75–140 pS. In both mutants, R↔C transitions (≤30 pS) were rare. Transitions >150 pS were sporadically observed in Cx43-CK1-D, extending the range of apparent channel amplitudes to values not present in Cx43-CK1-A and unreported for Cx43WT. At *V_j_* = 80 mV (>>*V_0_*), Cx43-CK1-D mostly displayed 35–55 and 75–120 pS transitions, and a few >150 pS transitions. In comparison, Cx43 CK1-A displayed 25–40 and 60–105 pS transitions, but essentially no >150 pS transitions. Note that while both mutants showed transition amplitudes above the typical 120 pS fully open state, they also displayed intermediate transitions compatible with a substate (80 > S > 40 pS), particularly at *V_j_* = 80 mV. Thus, in contrast to Cx43WT [14] both mutants preferentially displayed O↔C and O↔R transitions at small *V_j_* gradients, and O↔R and R↔C transitions at large *V_j_* gradients. On the whole, the data indicate that in the absence of *V_j_* gradients, mutant (and WT) channels may favor a fully open state. In addition, transition amplitude distributions from both mutants suggest a shift toward higher channel conductivity than Cx43WT.

### 2.3. Cx43-CK1-A Displayed Lower Permselectivity than Cx43-CK1-D

**Figure 3:**
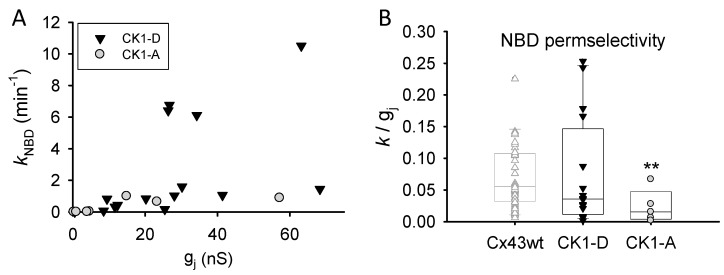
Permselectivity of dephospho-mimicking Cx43-CK1-A GJs is lower and less variable than that of phospho-mimicking Cx43-CK1-D GJs. (**A**) Rate constant of transjunctional dye diffusion vs. junctional conductance for the indicated mutants. Each symbol represents a single experiment; (**B**) Distribution (box plots) of collected permselectivity values for Cx43-CK1-D (0.074 ± 0.022; *n* = 16) and Cx43-CK1-A (0.017 ± 0.001; *n* = 7); Cx43WT data (from [14]) shown in light gray for comparison. Note that permselectivity values of Cx43-CK1-D (and Cx43WT) do not display a normal distribution. ** Median and variance of Cx43-CK1-A are different from Cx43-CK1-D and Cx43-WT (*p* < 0.05). See text for further explanation.

The appearance of highly conductive channels in the CK1-(de)phospho-mimicking mutants raised the possibility of these mutants displaying higher GJ permselectivity (molecular/atomic permeability: *P_j-_*_NBD_/*g_j_*) than Cx43WT [14,28]. Thus, NBD transjunctional diffusion (illustrated in Figure S3) and *g_j_* were measured in cell pairs expressing either mutant (see Section 4). Cx43-CK1-D expressing cells displayed (~three-fold) larger electrical coupling (*g_j_* = 30.86 ± 7.81 nS; *n* = 21) than those expressing Cx43WT (*g_j_* = 10.49 ± 1.48 nS; *n* = 53); however, permselectivity (Figure 3) of Cx43-CK1-D (0.074 ± 0.022 and Cx43WT (0.094 ± 0.018; [28]) expressing cells was similar. Cx43-CK1-A cells also displayed (~2 fold) greater electrical coupling (*g_j_* = 24.56 ± 8.79 nS; *n* = 12) than Cx43WT cells, but showed lower permselectivity (0.017 ± 0.001) than Cx43-CK1-D or Cx43WT (Figure 3), in agreement with previous studies [8]. It is noteworthy that the variability in permselectivity of Cx43-CK1-A was far less than either Cx43WT or Cx43-CK1-D (6% vs. 19% and 30%, respectively). These data suggest a link between Cx43 phosphorylation by CK1 and variable permselectivity; in contrast, low Cx43 permselectivity may follow dephosphorylation of the CK1 target serines.

### 2.4. Cx43-CK1-D and Cx43-CK1-A GJs are Resistant to Intracellular Acidification

**Figure 4:**
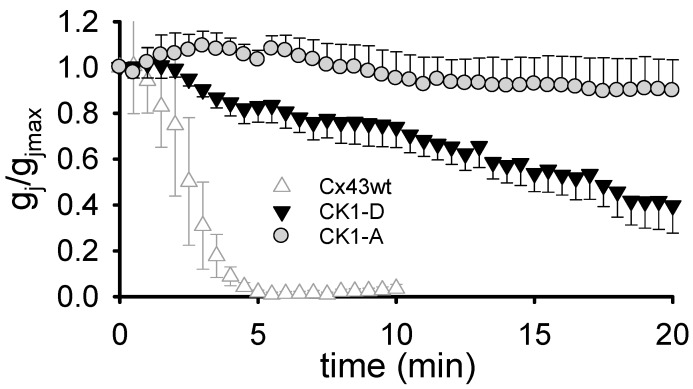
Cx43-CK1-D and Cx43-CK1-A mutants form gap junctions resistant to closure by low pH. Superfusion (starting at time 0) with a bicarbonate solution buffered at pH = 6.0–6.2 caused slow *g_j_* decrease in Cx43-CK1-D (black triangles; *n* = 6), and no *g_j_* decrease in Cx43-CK1-A (gray circles; *n* = 4) during an observation period of ≥ 20 min. The response of Cx43WT junctions to similar treatment (white triangles; *n* = 5) is reproduced from [14] for comparison.

In ischemic tissue, internal pH (pH_i_) falls to very acidic values (pH ~6.0) [29,30,31], and low pH-induced uncoupling can be arrhythmogenic [32,33,34,35]. To determine whether decreased pH-sensitivity of the Cx43 CK1-phosphoform confers the arrhythmia-resistant phenotype of S3E transgenic mice, we documented the response of Cx43-CK1-D *g_j_* to acidification. Unlike Cx43WT GJs, which typically close within ~5 min of exposure to low external pH (pH_o_), Cx43-CK1-D GJs remained open for > 5 min (Figure 4): ~54% and ~40% of initial *g_j_* lingered at 15 and 20 min, respectively, after the start of acidification. The overall delayed uncoupling reveals a major reduction of pH-sensitivity, suggesting that phosphorylation influences pH-gating of Cx43. In particular, phosphorylation by CK1 may decrease Cx43 susceptibility to low pH-induced uncoupling. Unexpectedly, however, the same acidification protocol also failed to uncouple Cx43-CK1-A GJs (Figure 4). Indeed, Cx43-CK1-A *g_j_* was ~93 and ~90% of initial at 15 and 20 min, respectively, and remained essentially unchanged following 25 min of low pH exposure, suggesting that dephosphorylation of CK1 targeted serines of Cx43 renders GJChs unable to close upon acidification. These data show that resistance of GJ coupling to acidification alone cannot explain the divergent cardiac phenotypes of transgenic S3A and S3E mice.

### 2.5. Cx43-CK1-D and -CK1-A Hemichannels Open Frequently

**Figure 5:**
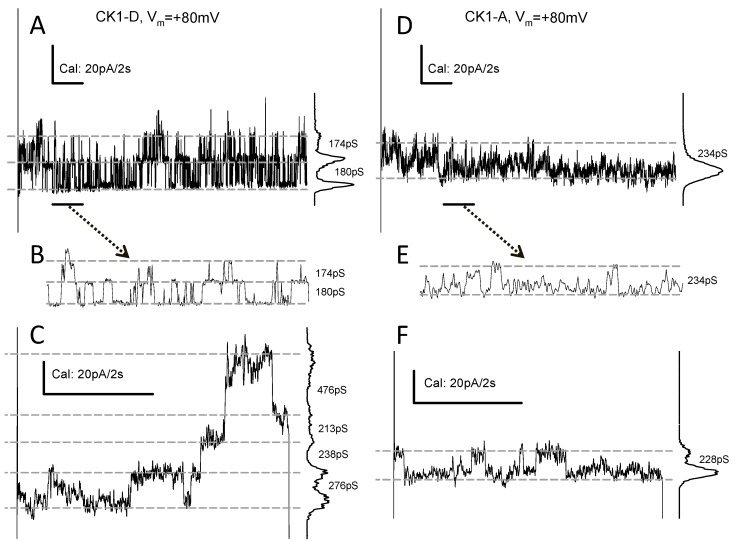
Cx43-CK1-D and Cx43-CK1-A expressing cells display frequent connexin HCh activity. Plasma membrane channel transitions compatible with opening of undocked connexons from single cells expressing Cx43-CK1-D (**A**–**C**) or Cx43-CK1-A (**D**–**F**). Lines, marks, plots, and numbers as in Figure 2. (**A**,**D**) are 20 s samples of longer recordings; (**B**,**E**) are expanded displays of the 2 s interval marked by black lines at the bottom left of (**A**,**C**); (**C**,**F**) Further examples of transitions recorded during 5-s pulses. Hemichannel activity from Cx43-CK1-D is usually well defined (as in **A**–**C**), in contrast to Cx43-CK1-A (**D**–**F**).

In Cx43WT-expressing cells at normal external calcium ([Ca^2+^]_o_) = 1–2 mM (and internal calcium, ([Ca^2+^]_i_) from nominally 0 to ~40 nM), connexin hemichannel (HCh) openings are rare and may require depolarization >+60 mV [36,37,38,39]. In Rin cells under our experimental conditions (see Section 4), this rarity is exacerbated, and few HCh-like transitions from Cx43WT have been recorded at *V_m_* = 80–100 mV (Figure S4). In contrast, membrane current (*I_m_*) transitions suggestive of HCh openings were seen in several, but not all cells transfected with Cx43-CK1-D (43 out of 127 explored, or ~33%) or Cx43-CK1-A (11 out of 62 explored, or ~18%). The amplitude of presumptive HCh transitions varied for both Cx43-CK1-D (140–280 pS; Figure 5A–C) and for Cx43-CK1-A (100–240 pS; Figure 5D–F). HCh activity of mutant Cx43-CK1-D varied from only a few events per second to profuse transitions (Figure 5A,B), not inactivated during >50 s pulses. In contrast, Cx43-CK1-A displayed less organized, harder to detect HCh activity (Figure 5D,E). Clear step-wise openings could be found in Cx43-CK1-D (Figure 5C), but less often in Cx43-CK1-A expressing cells. However, in three Cx43-CK1-A expressing cells, sudden *I_m_* increases suggesting multiple HCh openings, were seen during prolonged depolarizing pulses. One such *I_m_* increase (and recovery) occurred before the trace shown in Figure 5F, under well controlled *V_m_* clamp. The variability of HCh activities, in particular the infrequent appearance of Cx43-CK1-A events, encumbers wider comparisons between the mutants. Nevertheless, the records suggest that HChs formed by Cx43-CK1-D or Cx43-CK1-A can open under physiological conditions of internal and external Ca^2+^.

### 2.6. Cx43-CK1-D and -CK1-A are Phosphorylated at S368

**Figure 6:**
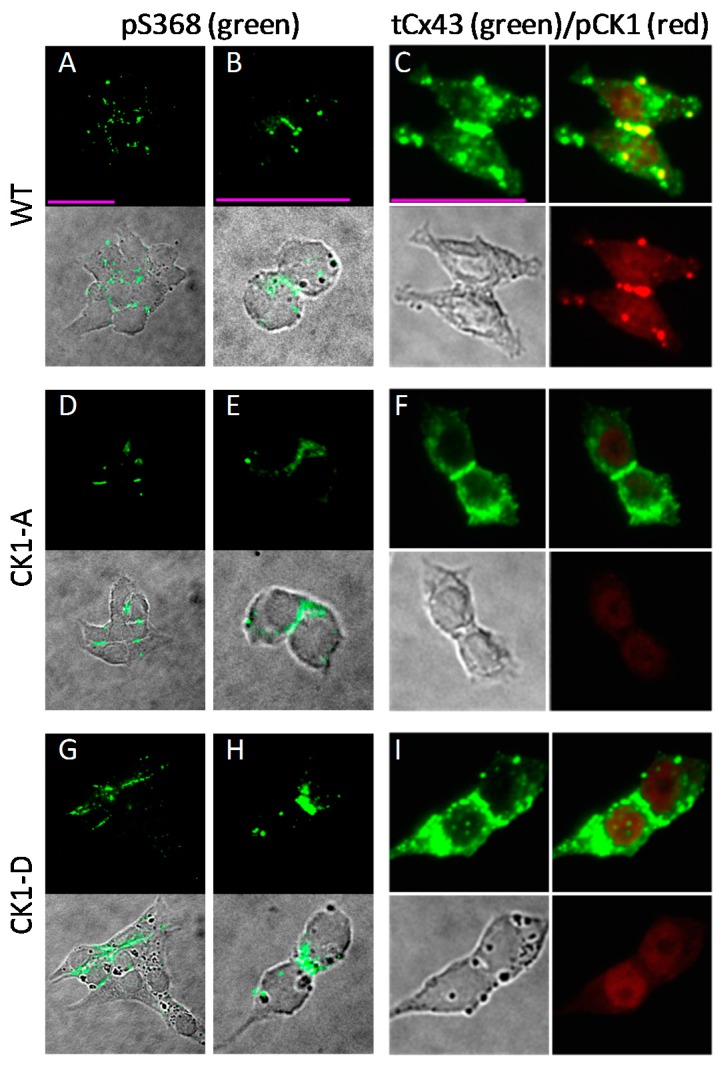
Residue S368 of Cx43 is phosphorylated in Rin cells expressing Cx43-WT, Cx43-CK1-A or Cx43-CK1-D. (**A**,**B**,**D**,**E**,**G**,**H**) Fluorescent (upper) and fluorescent merged with the corresponding differential interference contrast (DIC, lower) images of cells stained with a Cx43-pS368 phospho-specific antibody (pS368). Cx43-pS368 (green) was found in junctional plaques in groups and isolated pairs of Cx43WT (**A**,**B**), Cx43-CK1-A (**D**,**E**) and Cx43-CK1-D (**G**,**H**) cells. (**C**,**F**,**I**) DIC and fluorescent images of cells stained simultaneously with a polyclonal Cx43 antibody (“total” Cx43, tCx43) and a phospho-specific antibody against Cx43-p^CK1^ (pCK1). In all groups, total Cx43 (green) was found in junctional plaques and other cell areas (**C**,**F**,**I**, upper left panels). Bona fide CK1-phosphorylated Cx43 (red) was found only in Cx43WT cells (**C**, bottom right) colocalized with a membrane fraction of total Cx43 at junctional plaques (**C**, upper right, yellow). The pCK1 antibody labels a non-specific nuclear signal in the dephospho- (**F**) and phospho-mimicking (**I**) mutant expressing cells that is also found in parental Rin cells, devoid of connexins (Figure S5). Calibration bars (pink lines, 25 μm) apply to each column.

Our published data suggest PKC phosphorylation at residue S368 increases the dye permselectivity (*P_j-_*_NBD_/*g_j_*) of Cx43 GJs [28]. Hence, we pondered whether the discrepancy between Cx43-CK1-D and Cx43-CK1-A permselectivity might be consequential to differences in S368 phosphorylation. To address this possibility, we used phospho-specific antibodies to determine the presence and location of Cx43 phosphorylated at S368 (Cx43-pS368) in Cx43WT- and mutant-expressing cells. Representative images of this scrutiny show that the Cx43-pS368 epitope was present both at junctional (mainly) and non-junctional membranes, abundantly and in multiple spots in Cx43WT cells (Figure 6A,B). Although fewer cells in the Cx43-CK1-A clone were Cx43 positive, Cx43-pS368 signal was also present in this mutant (Figure 6D,E), with distribution comparable to Cx43WT. In Cx43-CK1-D expressing cells, Cx43-pS368 signal was readily found decorating long cell contacts (Figure 6G,H). Total Cx43 was profusely expressed, with a significant fraction located to junctional plaques, in all cell groups (Figure 6C,F,I, green). In addition, an antibody against CK1-phosphorylated Cx43 demonstrated the presence of this phosphoform in Cx43WT plaques, confirming that Rin cells are able to properly process and place this junctional protein (Figure 6C). These data demonstrate that phosphorylation of S368 occurs in both Cx43 mutants, but the data do not preclude the possibility that high permselectivity (in Cx43-CK1-D) is due to higher phosphorylation of S368 by PKC, and in turn, low permselectivity (in Cx43-CK1-A) results from lower pS368-Cx43.

## 3. Discussion

### 3.1. On the Role Cx43 of Phosphorylation

The cytoplasmic carboxyl terminus (CT) domain of Cx43 harbors several kinase consensus sites [5,40], but whether phosphorylation at these sites occurs independently or in tandem, transiently or continuously, alternately or progressively, remains unclear. Nevertheless, (de)phosphorylation at specific sites has been linked to specific Cx43 cellular locations [41,42,43,44,45,46,47]. How these phosphorylation differences and their associated structural modifications translate into functional changes in the context of cellular phenomena where Cx43 plays a role (e.g., cell cycle progression and proliferation [48,49,50,51], healing [52,53,54], migration [55,56,57,58]; differentiation [59,60,61] and electrical coupling [34,35,41,62,63]), remains undetermined. Here, we studied the electrical/chemical gating and the permselectivity of Cx43 (de)phospho-mimicking mutants associated to disparate cardiac phenotypes in transgenic mice (arrhythmia susceptibility vs. arrhythmia resistance). We found differences in channel function/regulation that may contribute to these cardiac phenotypes. Our data may be relevant to the pathophysiology of cardiac ischemia.

### 3.2. What the Data Suggest

#### 3.2.1. Voltage Gating and Channel States

Cx43-CK1-D and Cx43-CK1-A display differences in GJ *V_j_*-sensitivity and channel behavior. The fast exponential component of *V_j_*-gating may involve an interaction of the Carboxyl Terminus (CT) domain with a receptor-like structure near the pore mouth, formed by a region of the Cytoplasmic Loop (CL) [19,64,65]. Our *V_j_*-gating data suggest that CK1-phosphorylation enhances *V_j_*-sensitivity of Cx43 residing at the ID, and that the residual state would be more readily occupied by this phosphoform (Cx43-p^CK1^) when subjected to high *V_j_* gradients. In turn, the channel data suggest that at low *V_j_* gradients (the most likely environment of GJs), both the dephospho-(Cx43-dp^CK1^) and the phosphoform exist in either fully open or closed states, and at high *V_j_* gradients, both transit frequently between fully open and residual states, from which they can close.

Because at low *V_j_* and despite their charge differences, both mutants show larger channel transitions than Cx43WT, it is possible that high conductivity results from structural modifications of the mutated region rather than different charge polarity/density. Alternatively, the large unitary conductance (γ_j_) of mutant channels could reflect the “homomeric” nature of these channels (at least in regard to the CK1 targets) compared to Cx43WT, where differences in phosphorylation state between Cx subunits certainly must exist. Large γ_j_ values in mutants representing the extremes of Cx43 phosphorylation by CK1 cannot readily explain their opposite cardiac phenotypes. However, the Cx43-CK1-D data suggest that phosphorylation by CK1 enhances conductivity of Cx43 channels, as evinced by the larger O↔C and O↔R transitions (at low *V_j_*), and the wider residual state range (at high *V_j_*), than either Cx43-CK1-A or Cx43WT. Of note, oversized transitions were not observed in all Cx43-CK1-D cell pairs and did not establish a predominant γ_j_ peak, suggesting that CK1-phosphorylation alone is not sufficient to yield this highly conductive channel configuration. Another possibility is that large transitions are double channel events brought about by the high *V_j_*-sensitivity of the mutant, which cannot be discriminated under our recording conditions.

It is important to consider here the possible meaning of multiple channel transition amplitudes and their relative abundance. Such multiplicity is observed at *V_j_* < *V_0_* as well as > *V_0_*, in Cx43WT as well as Cx43-CK1-D and Cx43-CK1-A mutants. This multiplicity of transition amplitudes indicates that, in the absence of *V_j_* gradients, Cx43 channels reside stably (≥50 ms) in multiple open configurations, each of which can display *V_j_* sensitivity. If the only function of GJChs were to establish an electrical pathway between cells, the only relevant measure would be the absolute value of *g_j_*, irrespective of the multiple channel states that inhabit the junctional plaque. What, then, is the benefit of supporting multiple open states? It has been shown that electrical coupling and dye coupling are not linearly correlated [28,66]; perhaps we should ask whether and how multiple channel states regulate the intercellular flux of the “metabolites and second messengers” so often mentioned in the literature.

#### 3.2.2. pH-Gating

Susceptibility to acidification-induced uncoupling is a common trait of GJs, but pH-sensitivity varies among connexins [67,68,69,70]. As with *V_j_*-gating, a CT-CL interaction has also been proposed as the pH-gating mechanism [64]. Uncoupling of Cx43 GJs is quickly induced by superfusing cells with weak acid solutions buffered at pH_o_ ~6.0–6.2 [68,71], which pH_i_ readily follows [72]. In ischemic tissue, pH_i_ may fall to similar low values [29,30,31,73]. Therefore, during myocardial ischemia low pH_i_ could contribute to the decreased electrical coupling in damaged tissue, setting the stage for arrhythmogenesis [32,33,34,35]. In this scenario, delayed closure upon acidification and transient preservation of electrical coupling at the cells’ end-to-end GJs could temporarily protect impulse conduction, cardiac anisotropy, and metabolic rescue of failing cells, all favoring preservation of function early in an ischemic episode. However, during continuous ischemia, complete GJ uncoupling could limit tissue damage, to the detriment of the still living cells in the infarct area, but to the benefit of the organ. Thus, the transient resistance to acidification of Cx43-CK1-D would be consistent with the arrhythmia-resilience of S3E mice. On the other hand, persistent junctional coupling during prolonged acidification would negate the advantages of a transient preservation of coupling, by allowing the steady diffusion of noxious substances from the damaged area to surrounding tissue [32,33]. If this were the case, the imperviousness of Cx43-CK1-A to acidification-induced closure would be consistent with the arrhythmia-vulnerability of S3A mice, where increasingly larger lesions may facilitate arrhythmia. This possibility does not necessarily exclude TAC-induced arrhythmia susceptibility, a chronic condition where acute acidification is not a known factor, but where junctional permselectivity to regulatory molecules may play an important role.

#### 3.2.3. Channel Selectivity

The permselective variability of homomeric, homotypic Cx43 GJs has been linked to variable phosphorylation of the Cxs comprising the channels [28]. Results from Cx43-CK1-D and Cx43-CK1-A support the notion that permselectivity is an inherent property of Cx43 channels, not directly linked to the level of electrical coupling, but to the phosphorylation-induced functional states of the channels comprising the junction. Explicitly, CK1 phosphorylation may provide part of, or be permissive to, the permselective variability of Cx43 junctions. In turn, dephosphorylation of CK1 sites may be less permissive to, or limit (but not completely prevent) such variability. It was also proposed that decreasing permselectivity with unchanging *g_j_* was due to a decreasing proportion of highly permeable channels within the junction [74]. Our results from Cx43-CK1-A and previous data showing low dye (LY) coupling in mesenchymal cells expressing this mutant [8], are in agreement with that idea.

At this point, we should consider possible differences between the transgenic models and the fate of Cx43WT phosphoforms during pathological events. First, Cx43-CK1-A showed lower and less variable permselectivity (*P_j-_*_NBD_/*g_j_*) than Cx43WT, suggesting that despite its likely contribution to persistent electrical coupling (in the mouse model), this mutant’s ability to support transjunctional diffusion of organic ions (e.g., signaling molecules, nutrients, and waste products) may be limited. Whether this reduced permselectivity would make Cx43-CK1-A (or Cx43-dp^CK1^) in the infarct border zone more liable to propagating damage (bystander effect) or efficient in protecting non-injured tissue (metabolic rescue) requires further study. Interestingly, in transgenic mice with cardiac-selective Cx45 overexpression, increased arrhythmia vulnerability was linked to altered permselectivity (lower LY and higher biotin coupling) between myocytes [4], suggesting that positively charged junctional permeants in the molecular size range of biotin may be of interest in arrhythmogenesis.

Second, during ischemia (or pressure overload hypertrophy), Cx43WT dephosphorylated at the CK1 sites may be phosphorylated or dephosphorylated at other sites as it moves away from the IDs. Judging by its electrophoretic mobility alone, the Cx43-CK1-A mutant would appear overall as dephosphorylated [9]; but despite being a “CK1-dephosphoform” this mutant generates (electrically) stable GJs, even in conditions of low pH_i_, and therefore, may not accurately represent the Cx43-dp^CK1^. Possible functional effects of kinases targeting other Cx43 sites (see below) during ischemia and GJR, and the fate of Cx43-dp^CK1^ remain subjects of study.

#### 3.2.4. Hemichannels

Cardiac ischemia- and TAC-induced GJR involve an apparent mobilization of Cx43 from IDs to lateral membranes of myocytes [8,11]. It is uncertain whether this lateralized protein pool makes functional channels (as docked connexons or undocked HChs), but reports suggest that massive HCh opening is a pathophysiological occurrence in the aftermath of myocardial ischemia [75,76,77,78,79]. Cx43-CK1-D and Cx43-CK1-A HChs can display high activity in the presence of normal [Ca^2+^]_o_. To date, only one other Cx43 construct, Cx43*NT37 [14], has displayed robust HCh activity in our experimental settings, but this chimera, with exceptional inability to respond to several gating triggers, is an unnatural construct made to explore the interaction of Cx domains. In contrast, Cx43-CK1-D and Cx43-CK1-A emulate natural states of the protein. This raises the questions of whether the massive HCh opening during metabolic inhibition or ischemia is indeed linked to stable CK1-dependent Cx43 phosphoforms or, alternatively, the mutants’ HChs are artifactual. As shown previously [8,11], Cx43-p^CK1^ disappears from IDs during GJR. In contrast, CK1-A and CK1-D mutants seem to exist (and stay) in junctional and in non-junctional membranes ([9] and our current data), even in pathological conditions.

If the Cx43-CK1-D and Cx43-CK1-A mutants replicate phosphorylated states of Cx43WT, then the corresponding alternate Cx43 phosphoforms make HChs that open at different cellular locales. Thus, Cx43-p^CK1^ HChs would open toward the “gap” (the virtual space containing the extracellular regions of Cx43) or the perinexus, but it is unclear whether their opening would be consequential (e.g., modify the ionic composition of perinexus or cytoplasm; cf. [80]). In contrast, HChs made by Cx43-dp^CK1^ may open at the lateral membranes, where they would contribute to arrhythmia propensity by facilitating collapse of the membrane potential, decreasing (Na^+^ channel) excitability and mediating leak of substances into and out of the cells [75,81,82].

#### 3.2.5. Further Cx43 Phosphorylation

The cardioprotective S3E phenotype may be linked to a stable presence of Cx43 at the ID, and/or to a permissive/protective role of the mutation itself for phosphorylation at other Cx43 sites ([9], their Figure 1, Figure 2, Figure 3 and Figure 4). Without conflict with these interpretations, arrhythmia resilience might be partly due to the temporary resistance of the CK1-phospho-mimicking Cx43 mutant to low pH-induced gating.

Slowly migrating electrophoretic Cx43 bands, perhaps representing phosphorylated isoforms, are preserved in the S3E hearts [9]. This observation poses an interesting line of thought: In Cx43WT, dephosphorylated S365 (Cx43-dpS365) is permissive to S368 phosphorylation by PKC [83], an event linked to smaller γ_j_ and higher permselectivity [15,18,28] of the remaining active GJChs [50,84]. Because spironolactone and the S3E mutation yield parallel effects on Cx43 distribution and arrhythmia susceptibility [9,11], one can speculate that the Cx43-pS365 isoform, protected by the hormone inhibitor, is also protected in the transgenic S3E hearts. Were this the case, the rise of Cx43-pS368 and the shift toward smaller γ_j_ and higher GJ permselectivity would be deterred in the S3E hearts and in the CK1-D expressing cells. Our results partially concur with this possibility, as the CK1-D mutant displayed lower frequency of small GJCh transitions and no higher permselectivity values than Cx43WT. However, Cx43-pS368 was readily found in cells expressing Cx43-CK1-D, and less abundantly in Cx43-CK1-A expressing cells; high permselectivity was observed in some Cx43-CK1-D, but not in Cx43-CK1-A cell pairs. These data demonstrate that S368 remains a target of PKC in both Cx43-CK1-D and Cx43-CK1-A, but leaves the possibility open that differences between these mutants may be due to differences in the level of S368 phosphorylation. To address this issue, one possible strategy would involve Cx43 constructs carrying both the CK1- and PKC-(de)phospho- mimicking mutations.

Despite their possible drawbacks, and because of their relative ease, mutational techniques contribute importantly to the study of phosphorylation [85,86]. However, alternate mutant (mimicking) forms of single phosphorylation sites with outcomes equally differing from Cx43WT have been reported [87]. These and some of our data (e.g., single channel transitions, pH-gating) suggest that (de)phospho-mimicking amino acid substitutions may not recapitulate all features of biological (de)phosphorylation. It is also possible that similar outcomes indicate that the mutated residues are not absolutely essential, or that complementary or sequential changes at other sites are necessary, for the explored/expected outcomes. Thus, in addition to S368, PKC-phosphorylation of S262 was linked to cardioprotection and prevention of Cx43 lateralization in the context of preconditioning and reperfusion injury [88,89,90,91]. Also during ischemia, Cx43 can be phosphorylated at S373, which activates a 14-3-3 mode-1 binding domain in Cx43; pS373 may disrupt Cx43/ZO-1 interaction, while the complex Cx43/14-3-3 facilitates ubiquitination, internalization and degradation of Cx43 [84]. The role of phosphorylation in Cx43/ZO1 binding/release was recently further explored using (de)phospho-mimicking amino acid substitutions [87]. Results suggest that Cx43/ZO-1 interaction is set within a series of sequential and hierarchical phosphorylation/dephosphorylation steps that involve not only residue S373, but also S365, S368, S279/S282, S255, and S262. Specific phospho-antibodies against these and other important sites (e.g., S262, S279, S365) are not widely available. However, it would be interesting to explore the channel from (de)phospho-mimicking amino acid substitutions of all these sites.

#### 3.2.6. Implications of CK1-Phospho-Mimicking Mutants for a Channel Gating Model

Overall, the data shown here strongly suggest that the gating of gap junction channels cannot be explained with a simple/unique closure mechanism. The data also confirm that the CT domain plays a role in channel closure, and demonstrate that such a role is modified by phosphorylation. To illustrate these points, let us assume that the various triggers that cause GJCh closure shared a single gating mechanism. Because modifications to the mechanism itself must affect its response to any trigger, then, weak pH-gating, for instance, would be expected to match weak *V_j_*-gating. We showed strong *V_j_*-gating paired with weak pH-gating (opposite), and “WT-like” *V_j_*-gating paired with absence of pH-gating (opposite). Thus, in contrast to the chimera Cx43*NT37, which inhibited both pH- and *V_j_*-gating in the presence of an intact Cx43CT [14], the CK1-(de)phospho-mimicking mutations differentially modulate the response of GJs to gating triggers through Cx43CT. These observations can only be understood if the (CT-CL) interactions involved in *V_j_*- and pH-induced channel closure are dissimilar, at least for Cx43. In other words, while different triggers may share common elements, the specific molecular structures involved in electrical and chemical gating may not always be identical. Moreover, the availability and readiness of the gating elements are amenable to modification by phosphorylation, and thus by the changing cellular conditions.

## 4. Materials and Methods

### 4.1. Plasmid Construction

pcDNA3 neo plasmid (Thermo Fisher Scientific, Waltham, MA, USA) containing the rat Cx43 sequence was mutated at the N341 site to convert the amino acid sequence to mouse using the Stratagene’s QuikChange Lightning kit (Stratagene, San Diego, CA, USA) according to the manufacturer instructions and the following primers (mutations shown in bold): 341S F 5′ gatttccccgacgac**agc**cagaatgccaaaaag3′ and N341S R 5′ ctttttggcattctg**gct**gtcgtcggggaaatcg 3′. After verifying the sequence at the University of Arizona’s UAGC sequencing facility, mutations S325,328,330D (dubbed CK1-D) and S325,328,330A (dubbed CK1-A) were introduced also with the QuikChange kit (Stratagene, San Diego, CA, USA) and the primers (mutations shown in bold): CKIDx3 F 5′ catggggcaggccgga**gac**a ccatc**gac**aac**gat**cacgcccagccgttcg 3′ and CKIDx3 R 5′ cgaacggctgggcgtg**atc**gtt**gtc**gatggt**gtc**tccggcct gccccatg 3′; CK1-Ax3 F 5′ catggggcaggccgga**gc**caccatc**gc**caac**gca**cacgcccagccgttcg 3′ and CKIAx3 R 5′ cgaacggctgggcgtg**tgc**gtt**gg**cgatggt**gg**ctccggcctgccccatg 3′. pcDNA containing each mutation was amplified with the QIAgen Maxiprep kit (QIAgen, Hilden, Germany)) as per manufacturer instructions to produce material for transfection. Sequence was confirmed at the University of Arizona UAGC Sequencing Facility (Local University Services).

### 4.2. Cell Culture, Transfections and Protein Expression

Rat insulinoma (Rin) cells [51] were transfected with the Lipofectamine 2000 (Life Technologies, Grand Island, NY, USA) and pcDNA3 plasmid containing Cx43-CK1-D. Subclones were isolated by dilution cloning and tested for gene expression by Western blotting. GJ coupling was examined in subclones or recently transfected cells. Control experiments were performed in cells stably transfected with Cx43WT (Rin43). Protein expression of Cx43-CK1-D and CK1-A was confirmed by immunofluorescence and Western blot. Immunocytochemistry (ICC) was performed using primary antibodies against total Cx43 (Sigma-Aldrich, St. Louis, MO, USA), pS368-Cx43 (AbCam, Cambridge, MA, USA) and pS325/328/330-Cx43 (generous gift from Paul Lampe). Secondary antibodies (Jackson ImmunoResearch, West Grove, PA, USA) labeled with Alexa488 or Alexa647 were used for fluorescence detection. 

### 4.3. Electrophysiology

Electrical recordings were performed as described [14]. Briefly, junctional (*I_j_*) or membrane (*I_m_*) currents were recorded with square voltage pulses using dual or single whole-cell (WC) patch clamp and osmotically matched (300–330 mOsm) external (in mM: 142.5 NaCl, 4 KCl, 1 MgCl_2_, 5 Glucose, 2 Na-pyruvate, 10 HEPES, 15 CsCL, 10 TEA-Cl, 1 CaCl_2_), and pipette solutions (in mM: 124 KCl, 3 MgCl_2_, 5 Glucose, 9 HEPES, 9 EGTA, 14 CsCl, 9 TEA-Cl, 5 Na_2_-ATP, 0.5 CaCl_2_; calculated [92] free cytosolic Ca^2+^ ≤ 20 nM). Macroscopic junctional conductance (*g_j_*) was measured with repeated, 2-second transjunctional voltage (*V_j_*) pulses of ±10 mV. *V_j_*-gating was assessed with 5-s *V_j_* pulses of increasing magnitude from 0 to ±100 mV in 10 mV increments (step protocol) or with repeated pulses of ±80 mV (for *V_j_*-dependent fast *I_j_* inactivation). To quantify *V_j_*-gating, normalized *g_j_* (Gj = steady state/instantaneous *g_j_* (*g_j_*^ss^/*g_j_*^inst^)) values obtained with the *V_j_* step protocol were fit with a Boltzmann function, which depicts distribution of two channel states: maximally/minimally open, over the *V_j_* range [12,13,16]; from these G_j_/*V_j_* relationships, the following parameters were obtained: Gj_max_ and Gj_min_ (maximum and minimum normalized *g_j_*^ss^), *V_0_* (*V_j_* at which G_j_^ss^ is halfway between Gj_max_ and Gj_min_) and *z*, a value representing gating charges [12,13]. To quantify fast *I_j_* inactivation, the decrease of composite *I_j_* during repeated ±80 mV pulses were fit with exponential decays of 1st or 2nd order (cf. [13]). Channel transition amplitudes (γ_j_) were documented with *V_j_* = ±40 or 80 mV in poorly-coupled cell pairs with or without halothane treatment. Transitions (see Supplemental Figure S1) were considered to occur between fully open (O), residual (R) and closed (C) states; transitions with values intermediate between O↔R and R↔C point to the existence of a substate (S). To reveal the presence of hemichannels, repeated, 5-second or longer depolarizing pulses (*V_m_* = 80 mV, WC configuration) were applied to non-coupled or single cells. All-points histograms were made from short (5–12 s) fragments of extended recordings. As intracellular pH (pH_i_) closely follows the extracellular pH (pH_o_) when buffered with weak acid solutions [72], *g_j_* uncoupling was achieved by superfusing cells with bicarbonate-containing solution adjusted to pH = 6.0 to 6.4 (when bubbled with 95% CO_2_/5% O_2_).

### 4.4. Permselectivity

Measurements of the ratio of permeation to dye vs. current carrying ions (P_j-dye_/*g_j_*) were fully described [14,27]. Briefly, dyes NBD-m-TMA (NBD, a junctional permeant) and rhodamine-labeled 3000Da dextran (rhodex3000, unable to permeate junctions, Molecular Probes, Eugene, OR, USA) were delivered through a patch pipette in Whole Cell Voltage Clamp (WCVC) mode to one cell of a pair; total NBD fluorescence was timely imaged for up to 17 min or until NBD equilibrated in both cells; the second cell was then accessed in WCVC mode to document *g_j_*. For each pair, a rate constant (*k* ≡ junctional permeability to dye, P_j-dye_) representing the speed of transjunctional dye diffusion, was calculated and plotted vs. the associated *g_j_* (thus, permselectivity ≡ P_j-NBD_/*g_j_*). Rhodex3000 images (Molecular Probes, Eugene, OR, USA) and/or halothane-induced uncoupling helped to discard dye diffusion through cytoplasmic bridges.

### 4.5. Statistical Analysis

Analyses were performed in Excel (Office 2010, Microsoft Corp., Redmond, WA, USA), Origin (Version 7, OriginLab Corp., Northampton, MA, USA) and GraphPad Prism (Version 7, GraphPad Software, La Jolla, CA, USA). Values are reported as Mean ± SEM. Comparisons were performed with ANOVA and unpaired *t*-Test (significance at *p* < 0.05). For permselectivity and channel distribution, Kruskal-Wallis (ANOVA on ranks) and Mann-Whitney *U* test (significance at *p* < 0.05) were used. Graphics were created with SigmaPlot 2001 (Version 7.101, Systat Software, San Jose, CA, USA).

## 5. Conclusions

Our data suggest that phosphorylation of Cx43 by CK1 (normoxia) yields GJChs with strong *V_j_*-gating, large γ_j_ values, variable permselectivity, and resistance to low pH-induced uncoupling, all of which may be compatible with arrhythmia-resistance. In contrast, dephosphorylation of the CK1 sites (hypoxia) yields GJChs with near “wild type” (as seen in Rin cells) *V_j_*-gating, large γ_j_ values, low permselectivity and imperviousness to low pH-induced gating. These data suggest that persistently open GJChs may be deleterious during ischemia, despite (or because of) their low permselectivity. In addition, Cx43-dp^CK1^ HCh openings at the lateral membranes (rather than the ID) may worsen the arrhythmic propensity of the afflicted myocardium. While phosphorylation of S368 was shown in Cx43WT and in the CK1-(de)phospho-mimicking mutants, a role for pS368 levels in determining permselectivity remains possible. The data offer possible explanations for the cardiac phenotypes of S3A and S3E mice, and for the pathophysiological events that attend the development of ischemia- or TAC-induced GJR and arrhythmias. Gap junction channel gating cannot be described with a single closure mechanism; instead, the role played by the CT in channel closure can be modified by phosphorylation.

## Figures and Tables

**Table 1:** Boltzmann parameters of Cx43 WT and mutants.

Mutant	G_j max_	G_j min_	*V_0_* (mV)	*A*
Cx43WT	0.98/0.99	0.32/0.34	−64/+70	4.8/4.1
Cx43-CK1-D	1.1/1.0	0.21/0.34	−49/+46	12.1/5.4
Cx43-CK1-A	1.0/0.98	0.35/0.40	−71/+70	8.4/4.1

Boltzmann fit parameters are shown as rounded values for both negative and positive polarities. Exact values are reported on Table S2. Cx43WT values obtained from experiments are displayed in Figure S1.

## Supplementary Materials

Supplementary materials can be found at http://www.mdpi.com/1422-0067/19/6/1659/s1.

## Abbreviations

CK1	Casein Kinase 1
Cx	Connexin
Cx43	Connexin 43
CT	Carboxyl Terminus
CL	Cytoplasmic Loop
S3A	Transgenic mouse line expressing mutant Cx43-S325,328,330A
S3E	Transgenic mouse line expressing mutant Cx43-S325,328,330E
TAC	Transverse Aortic Constriction
GJR	Gap Junction Remodelling
ID(s)	Intercalated disc(s)
GJCh(s)	Gap Junction Channel(s)
HCh(s)	Hemichannel(s)
Rin	Rat insulinoma
WCVC	Whole Cell Voltage Clamp
pS	PicoSiemens
nS	nanoSiemens
pH_i_	internal pH
pH_o_	external pH
DIC	Differential Interference Contrast
CT	Carboxyl Terminus
CL	Cytoplasmic Loop
ZO-1	Zonula Occludens 1
