# Supplementary material for: Cx43 Channel Gating and Permeation: Multiple Phosphorylation-Dependent Roles of the Carboxyl Terminus
Source: Int J Mol Sci. 2018 Jun 4;19(6):1659. doi: 10.3390/ijms19061659 (PMC6032060; doi:10.3390/ijms19061659)
Supplement: Supplementary file 1 [file ijms-19-01659-s001.pdf]

International Journal of *Molecular Sciences*

MDPI

Article

Cx43 channel gating and permeation: multiple phosphorylation-dependent roles of the carboxyl terminus

Jose F. Ek-Vitorin, [1,*] Tasha K. Pontifex [1] and Janis M. Burt [1]

Supplemental Material

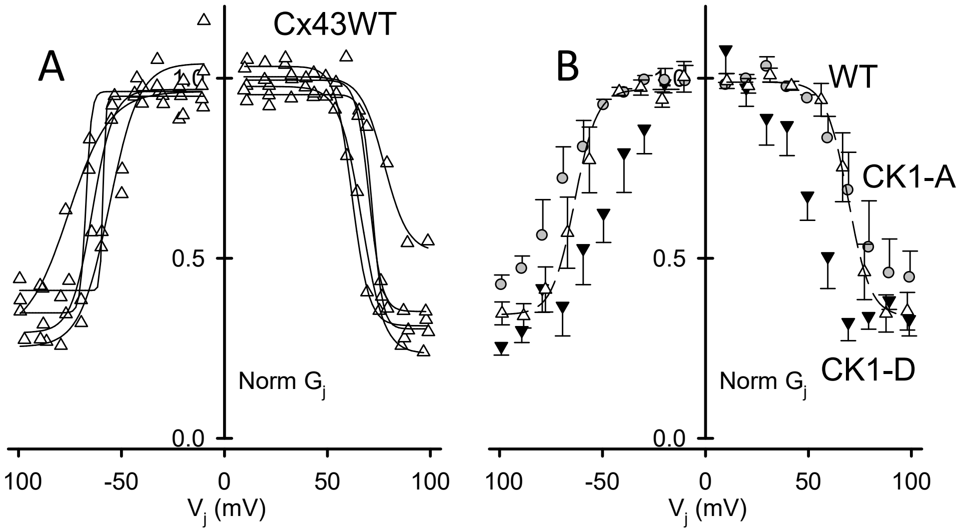

Supplemental Figure S1. Cx43-CK1-D displays stronger V_j gating than Cx43-CK1-A and Cx43WT. (**A**) V_j-dependence of G_j from individual experiments in Cx43WT-expressing cell pairs ($g_j = 4.1 \pm 1.1$; n = 5). Experimental values (white triangles) and their respective Boltzmann fits (black lines) are displayed. (**B**) Average V_j-dependence for Cx43WT (white triangles) and its Boltzmann fit (dashed lines). For clarity, Cx43-CK1-D (black triangles) and Cx43-CK1-A (gray circles) are reproduced here without Boltzmann fitting lines (cf. Figure 1). Cx43-CK1-D is different from Cx43-CK1-A and Cx43WT ($p < 0.05$; ANOVA, Tukey's post-hoc test).

Table S1. Time constants of fast Ij inactivation.

Mutant	τ_1 downward (s)	τ_2 downward (s)	τ_1 (upward (s)	τ_2 upward (s)
Cx43WT	0.21	--	3.80	0.40
	0.16	--	1.88	--
	0.26	--	0.27	--
	0.27	--	1.27	--
	0.66	0.08	1.14	0.20
Cx43-CK1-D	3.12	0.02	1.40	0.08
	1.58	--	0.25	--
	0.45	--	0.61	0.01
	0.20	--	0.44	--
	1.60	--	2.60	--
	1.44	0.13	4.40	0.26
	0.07	--	0.14	--
	0.10	--	0.52	--
Cx43-CK1-A	0.46	--	0.37	--
	8.70	--	1.63	--
	1.57	0.10	0.40	--
	0.18	--	8.70	0.40
	1.80	--	8.70	0.02

(--) τ_2 not detected

Supplemental Table S1. List of time constants (tau: τ) of I_j inactivation at V_j = ± 80 mV, for experiments in Figure 1 and Supplemental Figure 1. Ij was recorded at both polarities. Despite the small sample size, differences for all tau values from Cx43-CK1-D (0.92 ± 2.6, n = 21) vs Cx43-CK1-A (2.5 ± 0.99, n = 13) were significant (Two sample t-Test assuming equal variances, $p < 0.05$).

Table S2. Boltzmann fitting parameters from normalized g_j values.

Mutant	$G_{j\ max}$	$G_{j\ min}$	V_0 (mV)	*A*
Cx43WT	0.98 ± 0.01	0.32 ± 0.03	-64.4 ± 3.4	4.8 ± 1.8
	0.99 ± 0.01	0.34 ± 0.05	+69.7 ± 2.6	4.1 ± 0.6
Cx43-CK1-D	1.1 ± 0.05	0.21 ± 0.04	-49.2 ± 5.8	12.1 ± 4.1
	1.0 ± 0.05	0.34 ± 0.03	+46 ± 4.6	5.4 ± 1.5
Cx43-CK1-A	1.0 ± 0.04	0.35 ± 0.01	-71.1 ± 5.3	8.4 ± 3.9
	0.98 ± 0.02	0.40 ± 0.07	+69.9 ± 4.2	4.1 ± 0.6

Supplemental Table S2. List of Boltzmann fitting parameters for experiments shown in Figure 1 and Supplemental Figure 1. On either polarity, average V_0 was different between Cx43-CK1-D and either Cx43-CK1-A or Cx43WT ($p < 0.05$) but not between Cx43-CK1-A and Cx43WT (ANOVA followed by Tukey's post hoc test).

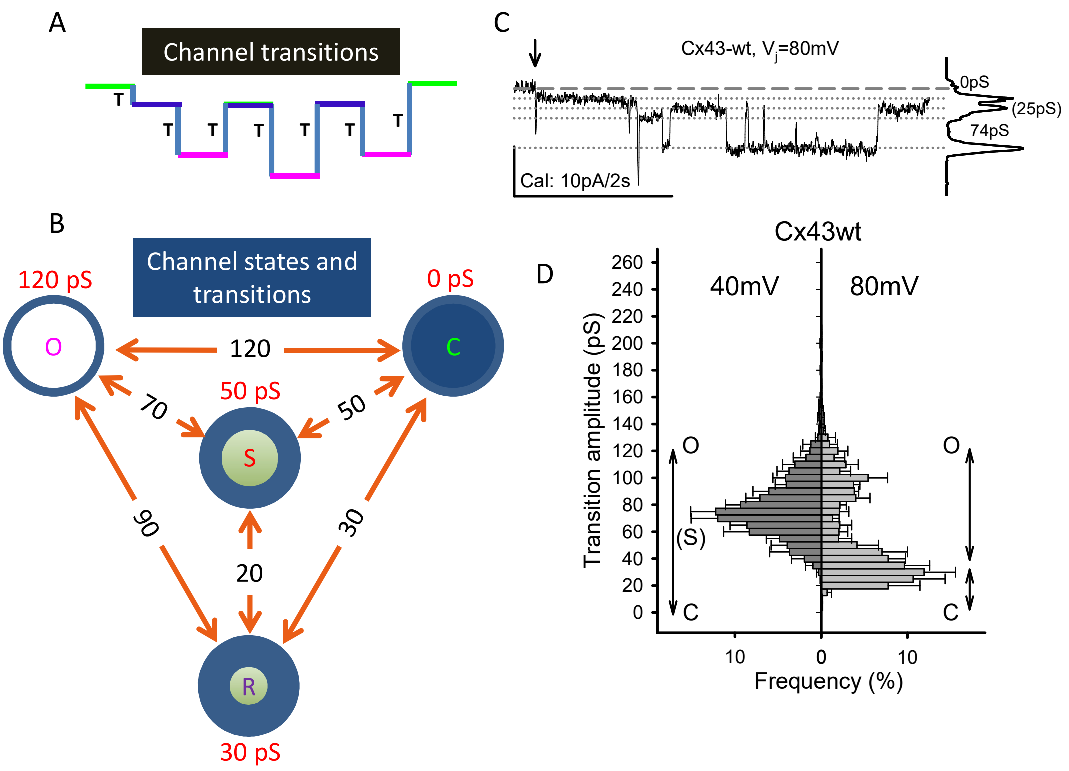

Supplemental Figure S2. Multiple junctional current transition values from Cx43WT GJChs indicate multiple channel conductive states. (**A**) Schematic of transitions between channel states. Transitions (**T**) of several amplitudes can be seen in channel recordings (when V_j is constant and $I_j \approx \gamma_j$). Notice that different I_j levels may be achieved with every transition. (**B**) Schematic of recognized channel states (labeled with their conductance, $\gamma j = I_j/V_j$) and I_j transitions between these states. GJChs exist in at least three different states, fully open (O), residually open (R) and closed (C), but an intermediate or substate (S) can be found. Reported γ_j values for these states (in pS, red text) and transitions between states (also in pS and denoted by red double arrowed lines, indicating reversibility; black text) are depicted. In this scenario, O↔C: 120, O↔R: 90 and R↔C: 30 pS. Transitions >30 and <90 pS suggest the existence of substates (S), conductive configurations different from fully open channel. (**C**) Typical recording of GJCh activity in Cx43WT-expressing cell pairs. Settings are described in the main text. (**D**) Frequency histograms of GJCh transitions collected from Cx43WT cells, at V_j = 40 mV (n = 12, N = 2386) and 80 mV (n = 5, N = 1124). Notice the non-Gaussian distribution at either V_j. A shift toward smaller values at 80 mV indicates that at this gradient channels transit more frequently between residual and closed states, than at Vj = 40 mV.

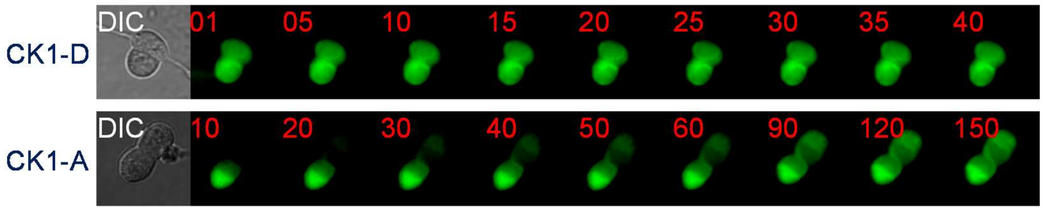

Supplemental Figure S3. Transjunctional dye diffusion between cells expressing either Cx43-CK1-D or Cx43-CK1-A. On each row, the injected (donor) cell is at the bottom and the recipient at the top; at the left, Differential Interference Contrast (DIC) images of the cell pair. From left to right, false colored (green) fluorescent images taken at times (seconds) shown. In these examples, brightness differences between donor and recipient can be detected at least within the first minute for Cx43-CK1-A (diffusion constant, k = 0.89), but differences are mostly obliterated within the first 10 seconds for Cx43-CK1-D (k = 10.5). Their g_j values (57 nS for Cx43-CK1-A and 63 nS for Cx43-CK1D) do not match their k differences.

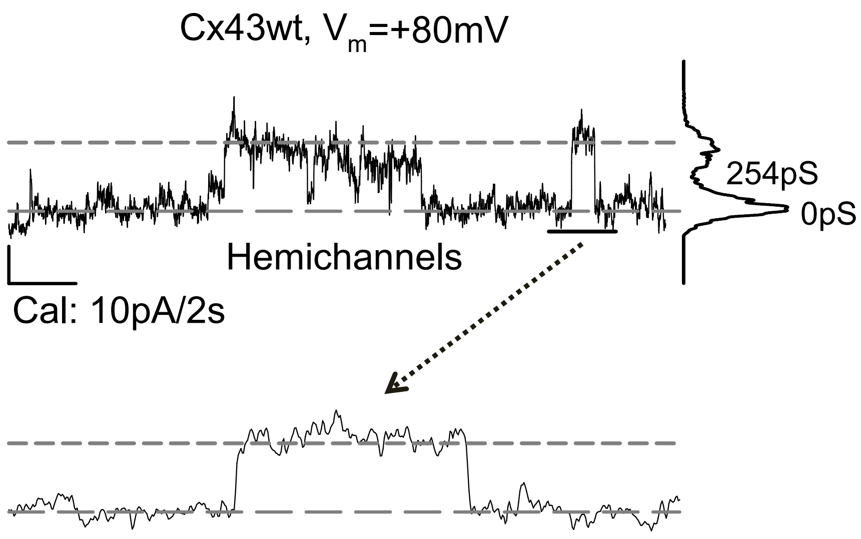

Supplemental Figure S4. Cx43WT HChs rarely open in Rin cells. This example shows transitions compatible with HCh openings (upper trace) and an expanded view of a two-second interval (lower trace). The events were documented in one of several long at V_m = 80 mV pulses. Total recording time: ~280 s.

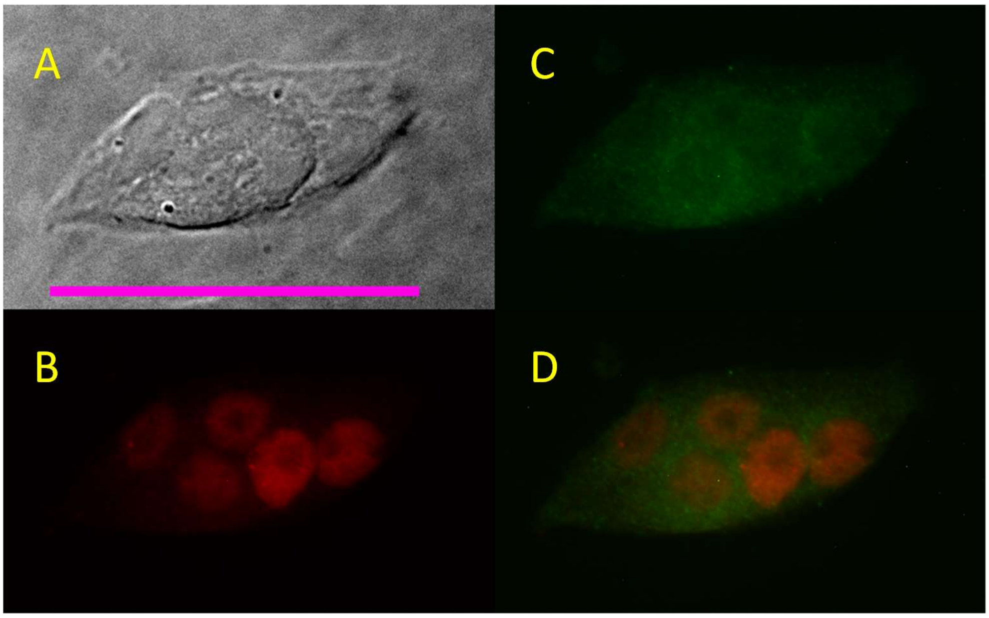

Supplemental Figure S5. The pCK1-Cx43 ab labels an epitope in the nuclei of parental Rin cells, which lack Cx43. (**A**) DIC image of a small group of Rin cells. (**B**) Unspecific nuclear staining by anti-pCK1-Cx43 ab. (**C**) Background staining with a total anti-Cx43 ab (see Methods). (**D**) Superposition of (**B**) and (**C**). Pink line in (A), Calibration: 50 µm.
